# Protocol for generating protoplasts of *Trichoderma koningiopsis* and transformation of plasmid DNA

**DOI:** 10.1016/j.xpro.2025.103668

**Published:** 2025-03-06

**Authors:** Sophie Jin, Fabrizio Alberti

**Affiliations:** 1 School of Life Sciences, University of Warwick, Gibbet Hill Road, Coventry CV4 7AL, UK

**Keywords:** Microbiology, Molecular biology, Biotechnology and bioengineering

## Abstract

*Trichoderma koningiopsis* is a filamentous fungus that produces numerous bioactive molecules and has the potential to be used as a biological control and plant growth-promoting agent. Here, we present a protocol for generating protoplasts of *T. koningiopsis*. We then describe four techniques for the stable transformation of plasmid DNA: polyethylene glycol (PEG)-mediated transformation of protoplasts, *Agrobacterium*-mediated transformation, electroporation of spores, and lipofection of mycelia. We then detail procedures for confirming the expression of a spCas9 gene using fluorescence microscopy.

## Highlights

• Protocol for the generation of protoplasts from *Trichoderma koningiopsis*
• Transformation techniques (PEG- and *Agrobacterium*-mediated) of *T. koningiopsis*
• Use of electroporation and lipofection as transformation techniques for *T. koningiopsis*

## Abstract (editor highlights)

Publisher’s note: Undertaking any experimental protocol requires adherence to local institutional guidelines for laboratory safety and ethics.

## Graphical abstract

**Figure:**
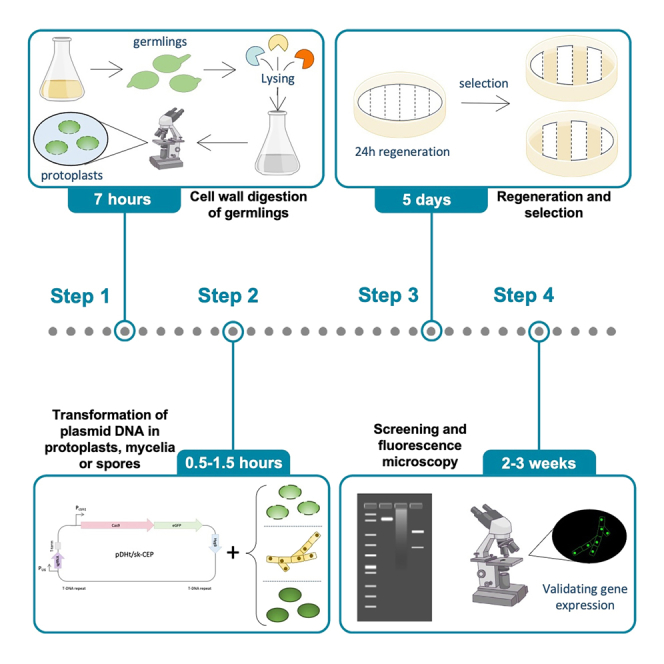

## Before you begin

Reports of transformation in *Trichoderma* spp. include *T. reesei*,^1^ *T. harzianum* and *T. atroviride*^2^^,^^3^ amongst others. Most successful modifications were made via PEG-mediated transformation of protoplasts and *Agrobacterium*-mediated transformation.^2^^,^^3^ Electroporation of protoplasts, biolistic of spores and the application of shock waves on spores were also attempted with success in *T. longibrachiatum*,^4^ *T. harzianum*^5^ and *T. reesei*^6^ respectively.

This protocol details several techniques for integration of exogenous DNA in *Trichoderma koningiopsis*, which is a known biocontrol agent of plant disease^7^^,^^8^^,^^9^^,^^10^^,^^11^^,^^12^ as well as the producer of bioactive specialized metabolites, including with antimicrobial and anti-inflammatory activity.^13^^,^^14^^,^^15^ This is the first report of a protocol for transformation in *T. koningiopsis* and will open possibilities for genetic modification of this economically relevant species. The approaches include PEG-mediated transformation of protoplasts, *Agrobacterium*-mediated transformation (ATMT), electroporation of spores and lipofection of mycelia. Protocols were adapted from existing protocols used for the transformation of other fungi.^2^^,^^3^

The transformation protocol can be conducted in one day for PEG-mediated transformation and ATMT, while for electroporation and lipofection, both can be done in a half day. However, earlier preparation of buffers and materials listed in the materials and equipment setup section is necessary. To prepare the cultures, the fungus must be grown between 3 to 5 days depending on the chosen technique. Mycelia can be produced in liquid culture after 3 days but for spores, the fungus must be grown on solid media for 4–5 days. After transformation, the fungus must be grown for 2 weeks, during which 3 subcultures are required to obtain homokaryotic transformants. Overall, the timeline for transformation ranges from 3 to 4 weeks. All experiments were carried out with the plasmid pDHt/sk-CEP, for which the backbone was obtained from Zhihua Zhou (Addgene plasmid #92126).^7^

**Figure 1:**
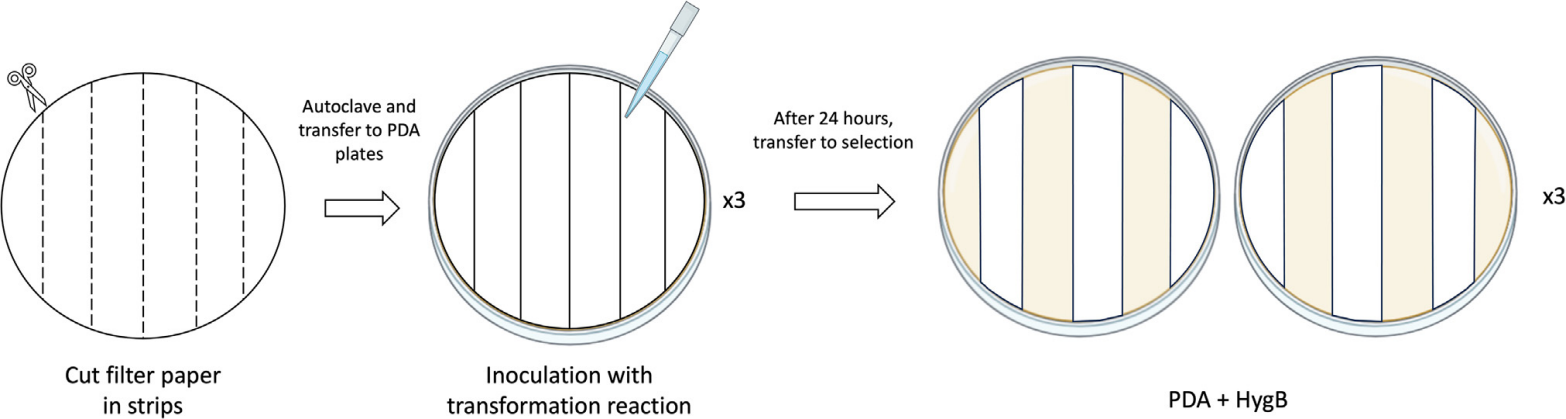
Schematic representation of the plating procedure for PEG-mediated transformation Filter papers (∅ 9 mm) are cut into 6 strips and autoclaved. They are subsequently transferred onto PDA plates to form PDA-strips plates where the transformation reaction is inoculated. After 24 h and for each plate, the strips are transferred to 2 PDA plates containing the selection marker.

### Media and buffer preparation

**Timing: 2–3 h**

1. For PEG-mediated transformation.
  a. Preparation of PDA solid medium at pH 6.2. With 2 plates, approximately 10^8^ spores/mL can be obtained.
  b. Preparation of CM liquid medium for production of germlings.
  c. Preparation of Protoplast buffer (PB) pH 7.5 and PEG buffer (PEGB) pH 7.5.
  d. Preparation of PDA-strips plates as shown in Figure 1.
2. For ATMT.
  a. Preparation of YEPAA solid medium for growth of *Agrobacterium tumefaciens* LBA4404.
  b. Preparation of IM liquid and solid medium.
  c. Autoclaving of nitrocellulose filters (0.8 μm pore size, Ø 47 mm).
  d. Preparation of PDA solid medium supplemented with 300 μg/mL cefotaxime and the selection marker.

**CRITICAL:** The pH of the IM medium needs to be adjusted to the strain of *A. tumefaciens*. Here, a pH of 5.8 for the IM was used to grow *A. tumefaciens* LBA4404.

3. For electroporation.
  a. Preparation of 1.1M sorbitol and YEPD liquid medium.
  b. Preparation of PDA selective plates.

**CRITICAL:** The sorbitol solution needs to be ice-cold when used to wash and resuspend the spores before transformation.

4. For Lipofection.
  a. Preparation of PDA-strips plates as shown in Figure 1.

***Note:*** Here, the selective marker used was hygromycin B at 150 μg/mL.

### Culture and collection of *T. koningiopsis* RA6 fresh spores

**Timing: 4–5 days**

5. *T. koningiopsis* RA6 spores are plated on 2 PDA plates pH 6.2 and grown for 4–5 days at 30°C in the dark (see troubleshooting 1).
  ***Note:*** Prepare the sterile glass funnels with Miracloth ahead of the harvest time point.
  a. To harvest the spores, pour 5–10 mL of 0.9% sterile saline solution onto each plate and detach from the plate using a sterile loop or L-shape spreader. The resuspended spores can then be filtered from mycelial fragments using Miracloth attached to a sterile glass funnel.
  b. Determine the spore concentration using a hemocytometer under the microscope. The final concentration should be no lower than 10^8^ spores/mL.
    **CRITICAL:** Transformations should be performed with fresh spores. For electroporation, spores can be flash frozen and kept at −80°C as aliquots for a few months. However, the best results will be with fresh spores. Spore viability can be checked prior to transformation by replating them on non-selective media.

## Key resources table

**Table:** 

REAGENT or RESOURCE	SOURCE	IDENTIFIER
**Bacterial and virus strains**		

*Trichoderma koningiopsis* RA6	Environmental isolate	Tamizi et al., 2022^12^
*Agrobacterium tumefaciens* LBA4404	Donated from Grant Lab (University of Warwick)	Ford et al., 2016^16^

**Chemicals, peptides, and recombinant proteins**		

D-sorbitol	MP Biomedicals	Cat#0210293805
DMSO 99.9%	Fischer Chemical	Cat#10162090
VinoTaste	Novozyme	
Chitinase from *Streptomyces griseus*	Merck Life Sciences	Cat#SAE0158
Driselase from Basidiomycetes sp.	Merck Life Sciences	Cat#D9515
Cas9 protein	Merck Life Sciences	Cat#CAS9PROT
Lipofectamine 2000 Transfection Reagent	Invitrogen	Cat#11668027
Cefotaxime	Merck Life Sciences	Cat#C7039
Acetosyringone	Merck Life Sciences	Cat#D134406
MES solution	Merck Life Sciences	Cat#76039
Triton X-100	Merck Life Sciences	Cat#X100

**Critical commercial assays**		

Master Mix PCR DreamTaq (2×)	Thermo Scientific	Cat#K1081

**Deposited data**		

*Trichoderma koningiopsis* RA6	NCBI	PRJNA786880

**Biological samples**		

pDHt/sk-CE	Addgene	Cat#92126
pDHt/sk-CEP	This study	Generated from pDHt/sk-CE, a sgRNA expression cassette was introduced within the T-DNA repeats under the control of an endogenous U6 promoter and T_8_-repeat terminator.

**Oligonucleotides**		

Tk_cas9_F (5′-CCCATCTTTGGCAACATT-3′)	IDT	NA
Tk_cas9_R (5′-ATGATGCGGGCATTGGAT-3′)	IDT	NA
Screen_gRNA_F (5′- CGCCAATATATCCTGTCA -3′)	IDT	NA
Screen_gRNA _R (5′-AGCTTGGATCAGATTGTC-3′)	IDT	NA

**Software and algorithms**		

ncbi-blast-2.13.0	NCBI	Altschul et al.^17^;https://blast.ncbi.nlm.nih.gov/Blast.cgi
Zeiss Zen v.3.5	Zeiss	https://www.zeiss.com/microscopy/en/products/software/zeiss-zen.html
Fiji v.1.0	ImageJ	https://imagej.net/software/fiji/
Benchling	NA	https://www.benchling.com/

**Other**		

1.5 mL tubes	Merck Life Sciences	Cat#HS4323
2 mL screw top tubes	Merck Life Sciences	Cat#HS10060
50 mL tubes	Merck Life Sciences	Cat#CLS430290
Whatman nitrocellulose membrane filter (∅ 4.7 cm, 0.8 μm pore size)	Merck Life Sciences	Cat#7188-004
Whatman filter paper (∅ 9 cm)	Merck Life Sciences	Cat#WHA1001090
Miracloth pore size: 22–25 μm	Merck Life Sciences	Cat#475855
Gene Pulser II electropolation system	Bio-Rad	Cat#165-2105
Electroporation cuvettes (0.1 cm gap)	Scientific Laboratory Supplies	Cat#Z706078
Square plates 12 cm × 12 cm	Merck Life Sciences	Cat#Z617679
T100 Thermal cycler PCR machine	Bio-Rad	Cat#1861096EDU
VelociRuptor V2 microtube homogenizer	Scientific Laboratory Supplies	Cat# SLS1401
L-shape spreader	Merck Life Sciences	Cat# HS8171A
ZEISS Axio Observer	Zeiss	https://www.zeiss.com/microscopy/en/products/light-microscopes/widefield-microscopes/axio-observer-for-materials.html

## Materials and equipment

**Table:** PDA

Reagent	Final concentration	Amount
Potato Dextrose Broth	24 g/L	24 g
Agar	20 g/L	20 g
Total		1 L

Autoclave (121°C for 15 min) and store at RT for up to 2 months.

**Table:** LB

Reagent	Final concentration	Amount
Tryptone	10 g/L	10 g
Yeast extract	5 g/L	5 g
NaCl	10 g/L	10 g
Total		1 L

Autoclave (121°C for 15 min) and store at RT for up to 2 months.

**Table:** CM

Reagent	Final concentration	Amount
Malt extract	5 g/L	5 g
Yeast extract	5 g/L	5 g
Glucose	5 g/L	5 g
Total		1 L

Autoclave (121°C for 15 min) and store at RT for up to 2 months.

**Table:** PWB pH 5.6

Reagent	Final concentration	Amount
KH_2_PO_4_	0.1 M	5.44 g
D-sorbitol	1.2 M	87.44 g
Total		0.4 L

Autoclave (121°C for 15 min) and store at RT for up to 2 months.

**Table:** PLB pH 5.6

Reagent	Final concentration	Amount
PWB	NA	20 mL
Vinotaste	30 mg/mL	600 mg
Driselase	15 mg/mL	300 mg
Chitinase	0.1 mg/mL	2 mg
Total		20 mL

Filter-sterilize and store at 4°C until use. Prepare fresh for each use to preserve lytic activity of the enzymes.

**Table:** PB pH 7.5

Reagent	Final concentration	Amount
Tris-HCl	0.01 M	2 mL of 1 M stock solution
D-sorbitol	1 M	36.43 g
CaCl_2_	50 mM	10 mL of 1 M stock solution
Total		0.2 L

Autoclave (121°C for 15 min) and store at RT for up to 1 month.

**Table:** PEGB pH 7.5

Reagent	Final concentration	Amount
Tris-HCl	0.01 M	1 mL of 1 M stock solution
D-sorbitol	1 M	36.43 g
CaCl_2_	50 mM	10 mL of 1 M stock solution
PEG6000	250 g/L	25 g
Total		0.1 L

Autoclave (121°C for 15 min) and store at RT for up to 1 month.

**Table:** YEPD

Reagent	Final concentration	Amount
Yeast extract	10 g/L	10 g
Peptone	20 g/L	20 g
Glucose	20 g/L	20 g
Total		1 L

Autoclave (121°C for 15 min) and store at RT for up to 1 month.

**Table:** YEPAA

Reagent	Final concentration	Amount
MgSO_4_.7H_2_O	0.5 g/L	0.5 g
Tryptone	5 g/L	5 g
Yeast extract	1 g/L	1 g
Beef extract	5 g/L	5 g
Sucrose	5 g/L	5 g
Agar	20 g/L	20 g
Total		1 L

Autoclave (121°C for 15 min) and store at RT for up to 1 month.

**Table:** IM pH 5.8

Reagent	Final concentration	Amount
KH_2_PO_4_	10 mM	1.74 g
K_2_HPO_4_	10 mM	1.36 g
NaCl	10 mM	0.58 g
MgSO_4_.7H_2_O	0.6 g/L	0.6 g
CaCl_2_.2H_2_O	10 mg/L	10 mg
KNO_3_	0.5 g/L	0.5 g
Glycerol	5 g/L	5 g
Glucose	2 g/L	2 g
FeSO_4_	1 mg/L	1 mg
2-(N-Morpholino)ethanosulfonic acid (MES)	40 mM	7.81 g
Acetosyringone (AS)	200 μM	To add for each culture from 1000× stock solution (39.2 mg in 1 mL for 200 mM stock)
ZnSO_4_.7H_2_O	0.5 mg/L	Make a 1000× stock solution containing all the trace metals and keep at 4°C. Add 1 mL of the stock **after** autoclaving.
CuSO_4_.5H_2_O	0.5 mg/L	
MnSO_4_.H_2_O	0.5 mg/L	
NaMoO_4_.7H_2_O	0.5 mg/L	
H_3_BO_3_	0.5 mg/L	
Total		1 L

Autoclave (121°C for 15 min) and store at RT for up to 1 month. Add AS and trace metals after autoclaving.

## Step-by-step method details

### Generating protoplasts from spores

**Timing: Incubation time to generate germlings (13–15 h)**

This step describes how to digest the cell wall of *T. koningiopsis* to obtain protoplasts from a spore suspension. This will enable transformation of the cells with exogenous DNA.

1. Germination of *T. koningiopsis* spores.
  a. From the spore suspension obtained in the previous steps, inoculate 100 mL of CM liquid medium with 5 × 10^8^ spores in a sterile 500 mL shake flask.
  b. Incubate the flask at 30°C, 170 rpm for at least 13 h, and up to 15 h.

***Note:*** The germination time can vary under laboratory conditions and a first assay should be carried out where monitoring of the germination is done under the microscope every 2–3 h.

**CRITICAL:** Finding the optimal germination stage is critical for effective lysis of the cell wall. The optimal state of germination can be determined by microscopy and germlings should look like engorged spores with budding mycelia.

**Figure 2:**
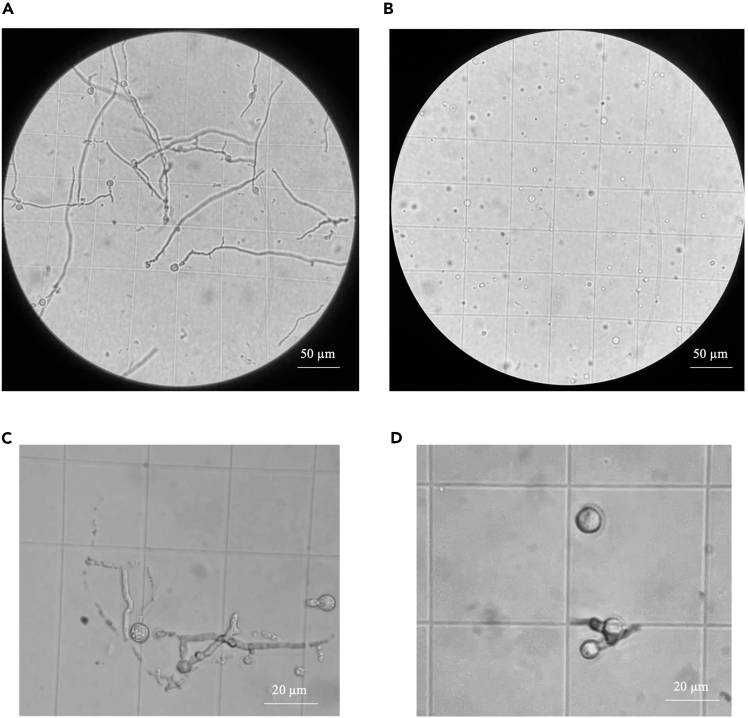
Digestion of germlings from *T. koningiopsis* RA6 using a lysing enzyme mix Germlings were incubated for 3 h 30 min and microscope pictures were taken at 30 min (A, C) and 3 h 30 min (B, D). Pictures were taken at x200 (A, B) and x400 (C, D). Fully digested protoplasts can be seen in panels B and D.

2. Collect the germlings from the culture by filtering the culture through Miracloth.
  a. Wash the retentate with 5 mL of PWB twice.
3. Keep the retentate and discard the flow through. From the retentate, resuspend around 0.5 g of germlings into 20 mL of PLB.
  a. Incubate at 30°C at 60 rpm (gentle shaking) for 3 h 30 min (see troubleshooting 2).
  b. Check the integrity of the protoplasts under the microscope every hour (Figure 2).

**CRITICAL:** If the cells appear deformed, the concentration of the osmotic stabilizer (sorbitol) needs to be adjusted. Other stabilizers can also be used like MgSO_4_ or NaCl.

4. Collect the protoplasts by filtering the 20 mL of lysate through Miracloth and keep the flow through. From this point the protoplasts need to be kept **on ice**.
5. Check the concentration of the protoplast using a hemocytometer. Centrifuge the protoplast solution at 3,200 × *g*, 4°C for 10 min or until the cells pellet.
6. Remove the supernatant being careful not to disrupt the pellet and resuspend the cells in PB to reach a concentration of >10^7^ protoplasts/mL.
7. Divide the solution into 200 μL aliquots and stored in sterile 1.5 mL tubes. Each aliquot can be used directly for transformation or can be stored up to 6 months at −80°C.

### Preparation of *Agrobacterium tumefaciens* LBA4404 cells for ATMT

**Timing: 3 days**

This step describes the preparation of the *A. tumefaciens* cell suspension containing the vector for transformation using ATMT.

8. Grow the *A. tumefaciens* strain containing the DNA vector of interest on YEPAA at 30°C for 48 h with the appropriate antibiotic selection.
9. Transfer a single colony from the plate to 10 mL of LB medium with the appropriate antibiotic selection and grow at 30°C overnight (see troubleshooting 3).
10. Centrifuge the culture at 3,200 × *g* for 10 min to collect the bacteria.
11. Resuspend the pellet in IM medium to reach OD_600_ = 0.1–0.2 using a spectrophotometer.

***Note:*** A negative control without AS should be prepared to check for the necessity of the virulence genes in the transformation.

12. Incubate the suspension at 30°C, 170 rpm until the OD_600_ = 0.4–0.5.
13. Concentrate the culture to half volume and the suspension can be used for ATMT.

**CRITICAL:** The pH of IM needs to be adjusted to each strain of *A. tumefaciens*.

### Preparation of mycelial suspension for transformation by lipofection

**Timing: 3 days**

This step describes the preparation of a mycelial suspension for lipofection. This approach is to be considered for transformation in strains which do not sporulate.

14. *T. koningiopsis* is grown from −80°C stocks in 5 mL PDB in glass universals at 30°C for 2–3 days.
15. About 0.1 g of mycelium in suspension is transferred in a 2 mL screw top tube containing bashing beads (∅ 0.5–1 mm).
16. The mycelia are then disrupted using a VelociRuptor with the following settings: 1 cycle of 4 m/s for 40 s.
17. The resulting suspension must be kept on ice and can be used for lipofection.

### PEG-mediated transformation

**Timing: 1–2 h**

This step describes the use of PEG to transform protoplasts.

18. For each transformation reaction, use one protoplast aliquot from step 7.
19. Add >5 μg of vector DNA solution along with 50 μL of PEGB and mix gently. Incubate for 20 min on ice (see troubleshooting 4).
20. Add 2 mL of PEGB, mix gently and incubate for 25 min at RT.
21. Add 3 mL of PB, mix gently and incubate for 5 min at 25°C.
22. Pour the transformation mixture onto 5 PDA-strips plates and incubate overnight at 30°C.
23. Transfer the strips to selective plates as shown on Figure 1 and incubate at 30°C for at least 5 days. Here, up to 8 colonies were observed on the plate after 5 days.

### Electroporation of spores

**Timing: 1–2 h**

This step describes the use of electroporation to introduce exogenous DNA into *T. koningiopsis* spores. This protocol was adapted from the patent from Kim et al., 2010.^18^

24. Culture *T. koningiopsis* as described in the before you begin section step 5.
  a. Harvest spores by pouring 5 mL of **ice cold** 1.1 M sorbitol.
  b. Filter the resuspended spores from mycelial fragments using Miracloth attached to a sterile glass funnel.

***Note:*** From here, the spores need to be kept **on ice** unless specified otherwise.

25. Spore concentration must be established using a hemocytometer and adjusted to 10^8^ spores/mL.
26. Divide the spore suspension into 200 μL aliquots in sterile 1.5 mL tubes.

***Note:*** The aliquots can be stored at −80°C for up to 6 months.

27. Add >5 μg of vector DNA to 200 μL of spores, mix gently and transfer into a 0.1 mm electroporation cuvette. Keep on ice for 5 min.
28. Electroporate at 16 kV/cm voltage and 25 μF capacitance (see troubleshooting 5).
29. After electroporation, transfer in 5 mL of a 5:1 mixture of 1.1 M sorbitol and YEPD liquid medium and incubate overnight at 30°C for regeneration.
30. Transfer the whole overnight culture onto 12 cm × 12 cm PDA plates containing the appropriate selection marker. Grow at 30°C for at least 5 days. Here, up to 10 colonies were observed on the plate after 5 days.

### *Agrobacterium*-mediated transformation

**Timing: 1–2 h**

This step describes the use of *Agrobacterium tumefaciens* to transform spores.

31. Mix 200 μL of *A. tumefaciens* suspension (step 13) with 200 μL of spores (step 5b of the before you begin section).
32. Spread the mixture onto IM agar plates overlaid with a nitrocellulose filter.
33. Incubate for 7 days at 30°C.
34. Remove the filter from the IM plate and place it on PDA plates with 300 μg/mL cefotaxime and the appropriate selection marker. Grow at 30°C for at least 5 days. Here, up to 15 colonies were observed on the plate after 5 days.

### Lipofection of mycelia

**Timing: 1–2 h**

This step describes the use of lipofectamine on mycelia for transformation of *T. koningiopsis*. The vector DNA can be encapsulated into liposomes using lipofectamine and introduced into fungal mycelia using this approach. This is an advantageous technique for strains which do not sporulate.

35. Mix >10 μg of vector DNA with 50 μL of lipofectamine 2000 and place the mixture on ice for 30 min.
36. Add 1 mL of the chilled mycelial suspension from step 17 to the liposomes and place on ice for a further 30 min.
37. Pour the resulting transformation reaction onto PDA-strips plates (Figure 1) and incubate overnight at 30°C. No growth should be observed from this step.
38. Transfer the strips to selective plates as shown in Figure 1 and incubate at 30°C for at least 5 days. Here, up to 10 colonies were observed on the plate after 5 days.

### Producing mitotically stable lines

**Timing: 2–3 weeks**

For all the transformation techniques, the transformants will appear after around 5–7 days. The transformants need to be subcultured under selective pressure 3 times before being considered homokaryotic.

39. After 5–7 days, transfer each colony onto a new PDA selective plate (see troubleshooting 6).
40. After 4 days, repeat step 37.
41. Repeat step 38.

### Validation of genome integration

**Timing: 1 day**

Once the transformants are homokaryotic, screening for plasmid integration can be performed. This is done using PCR on genomic DNA. The gDNA can be extracted from mycelia using the protocol previously described by Liu et al., 2000.^19^

42. Extract the genomic DNA as per Liu et al., 2000.^19^
43. To check for integration, the following PCR mix and cycles can be used with the appropriate primers. Here Tk_Cas9_F / Tk_Cas9_R and Screen_gRNA_F / Screen_gRNA_R were used to screen for integration of the plasmid pDHt/sk-CEP.

**Table:** PCR reaction master mix

Reagent	Amount
gDNA	1 μL
DreamTaq 2× Mastermix	12.5 μL
Forward primer	1 μL (1 μM)
Reverse primer	1 μL (1 μM)
ddH_2_O	To 25 μL

**Table:** PCR cycling conditions

Steps	Temperature	Time	Cycles
Initial Denaturation	95°C	3 min	1
Denaturation	95°C	30 s	25–40 cycles
Annealing	45°C^a^	30 s	
Extension	72°C	30 s^b^	
Final extension	72°C	5 min	1
Hold	4°C	Forever	

a The annealing temperature depends on the primer pair and needs to be adjusted.

b The elongation time needs to be adjusted depending on the length of the target and the rule of 30 s/kb can be used with this kit.

**Figure 3:**
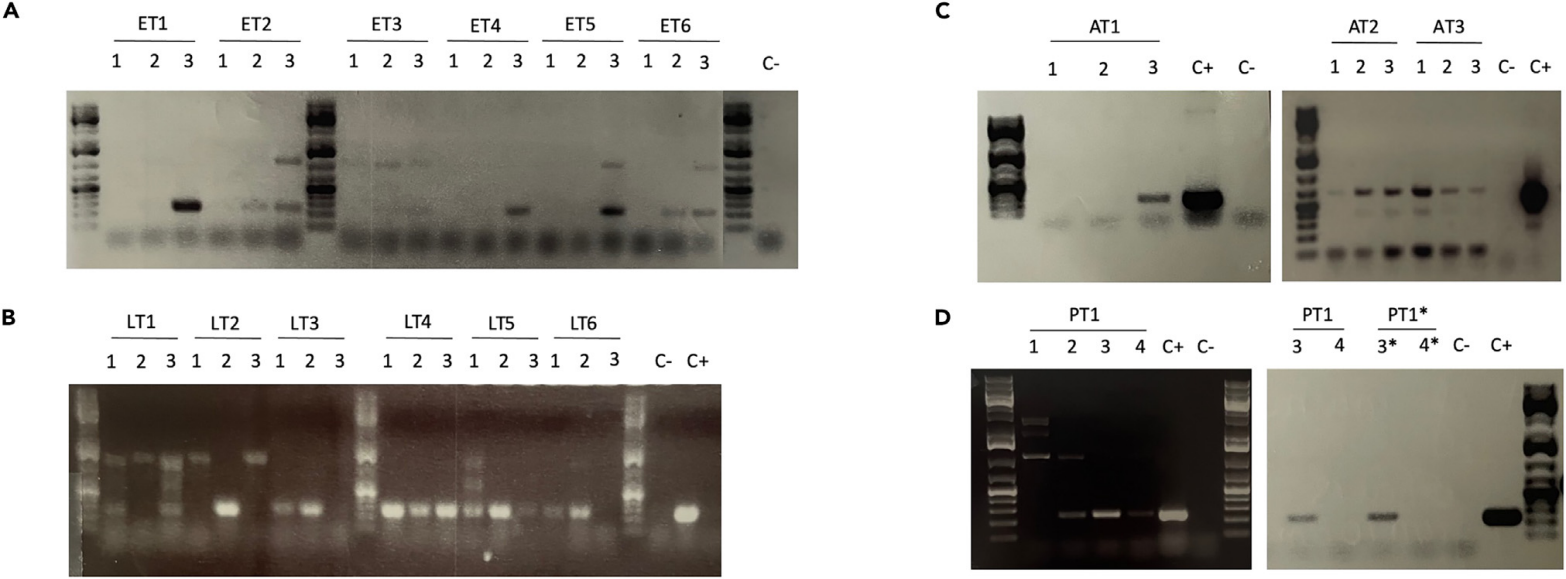
Screening for correct integration of pDHt/sk-CEP in *T. koningiopsis* RA6 genomic DNA Each subculture (1, 2, and 3) has been screened using Tk_Cas9_F/Tk_Cas9_R (A–D) and Screen_gRNA_F / Screen_gRNA_R for the transformant obtained with ATMT (B). Electroporation (A), ATMT (B), Lipofection (C) and PEG-mediated transformation (D) each yielded correct integration of the plasmid. The positive control (C+) used was pDHt/sk-CEP and the negative control (C-) was nuclease-free water. Expected band sizes: 243 bp for Tk_Cas9_F / Tk_Cas9_R and 683 bp for Screen_gRNA_F/Screen_gRNA_R.

44. Visualize the PCR products on a 0.1% agarose gel (45 min at 100 V). Results of correct integration can be seen in Figure 3.

### Validation of gene expression using reporter gene and fluorescence microscopy

**Timing: 7 days**

Once correct integration has been verified via PCR, the transformants can be cultured in MM2 with 0.1% (w/v) lactose to induce expression of the eGFP to validate gene expression. Indeed, the Cas9::eGFP transcriptional fusion in pDHt/sk-CEP is under the control of P_cbh1_, an lactose-inducible promoter.

**Figure 4:**
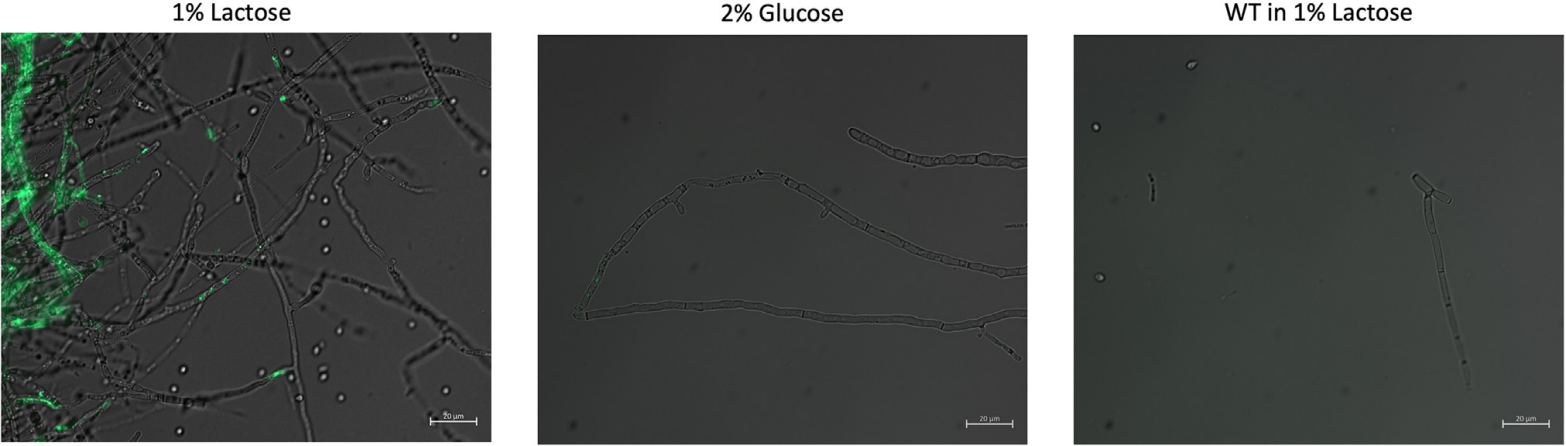
Fluorescence microscopy on *T. koningiopsis* RA6-CEP for validation of Cas9::eGFP expression when induced with 1% (w/v) lactose Images were taken from 7-day-old mycelia with ZEISS Axio Observer under blue light and overlaid to bright-field images. *T. koningiopsis* RA6-CEP fluorescence is compared under inducing conditions (1% (w/v) lactose), repressive conditions (2% (w/v) glucose) and with *T. koningiopsis* RA6 wild type (WT).

45. Culture the positive transformants in 20 mL of liquid MM2 with either 0.1% (w/v) lactose or 0.2% (w/v) glucose for 7 days at 30°C, 170 rpm.
46. Disrupt the mycelia as per steps 15–17. The resulting suspension is placed onto a microscope slide and observed under a fluorescence microscope.
47. Gene expression can then be seen via fluorescence under blue light as seen in Figure 4.

## Expected outcomes

Optimal germination can be obtained after 13–15 h of culturing in CM liquid medium. If using a different medium, the results will vary. Germination can be achieved quicker in PDA, but careful monitoring is necessary to not over-germinate. For each transformation, between 3 and 15 transformants are typically obtained and are detailed in the step-by-step protocol. *Agrobacterium*-mediated transformation yielded the most colonies after selection (15) while electroporation of spores gave the highest rate of positive transformants within the colonies obtained after transformation (45%) which brings the overall integration efficiency for this method to ∼1 CFU/μg of DNA.

## Limitations

The four different transformation procedures described here are established in *T. koningiopsis*. Each approach can be adjusted depending on the strain or species used but modifications will be necessary for germination, cell wall lysis and timings. Particularly in the case of PEG-mediated transformation, the viability of the protoplasts is the main source of limitations towards transformation efficiency. In this instance, a viability assay was conducted to assess the survival rate of the protoplasts and issues with regeneration were observed. In this case, switching the osmotic stabilizer and increasing its concentration helped getting higher rates, and low regeneration rates were found to hinder transformation efficiency greatly.

Markers other than hygromycin B resistance can be used, such as auxotrophic markers but strains with validated auxotrophy must be generated first if not already available. Potential issues may arise if the cells are not in the correct physiological state for the specific transformation protocol to be used. For instance, the preparation of spores for transformation *via* electroporation, PEG-mediated transformation of protoplasts and *Agrobacterium*-mediated transformation, can only be achieved if the fungi are grown until stationary phase is achieved. Similarly, the germination of spores into germlings to be used for PEG-mediated transformation of protoplasts should not be prolonged beyond the recommended time (and shall be monitored *via* microscopy) as this could lead to the production of mature mycelia that are associated with lower transformation efficiency compared to young germlings. In all cases, timings of growth and spore germination must be adapted to the specific strain used and carefully monitored.

## Troubleshooting

### Problem 1

The concentration of spores obtained from the solid cultures on PDA is too low.

### Potential solution

If the number of spores is too low, exposure to light can increase spore production in *Trichoderma*.

### Problem 2

The spore cell wall is not fully digested and no transformants are seen on the selective plates.

### Potential solution

If the cell wall is not fully digested even after 4 h, the concentration of each lytic enzyme can be adjusted. Increase in the concentration can help improve digestion, or swapping enzyme with others like lyticase or zymolase can also help. As the lytic enzymes mix from *Trichoderma harzianum* has been discontinued from Merck, a combination of a few others might be necessary to achieve full digestion. Another solution is to reduce the mass of starting material (mass of germlings) or incubate overnight instead.

### Problem 3

The *A. tumefaciens* culture is forming visible clumps in LB when cultured overnight.

### Potential solution

Some of the culture conditions can be changed to avoid clumping. Swapping LB for YEP (20 g/L Bacto peptone, 10 g/L yeast extract) or YEM (1 g/L yeast extract, 10 g/L mannitol, 0.5 g/L K_2_HPO_4_, 0.2 g/L MgSO_4_, 0.1 g/L NaCl) can reduce clumping, reducing the temperature can also help. Traces of contamination by other bacteria or phages can also cause clumping due to cell lysis.

### Problem 4

No transformants are seen after transfer to selective plates.

### Potential solution

Increasing the amount of vector DNA for each transformation can help increase yield. Linearizing the plasmid with a restriction enzyme can also increase integration. The addition of 0.006% Triton X-100 to further permeabilize the membrane for increased transformation efficiency.

### Problem 5

An electric arc can be seen when electroporating.

### Potential solution

Some of the reactions may produce an electric arc when subjected to the electric field. This might be due to a high concentration of salts if using MgSO_4_ or NaCl as an osmotic stabilizer, a high concentration of salt in the buffer of the DNA if not using nuclease-free water or TE buffer, a high concentration of cells, an excessively high voltage during electroporation, or residual moisture on the electrodes outside of the cuvette. Using less cells or exploring a range of different voltages can increase transformation efficiency.

### Problem 6

Colonies can be seen in the negative control where no DNA was introduced.

### Potential solution

If colonies appear in the negative control, the problem might be that the concentration of the antibiotic used is too low or that contamination with other fungi was introduced. Screening the resistance of the strain prior to transforming or the use of a different selective marker is recommended.

## Resource availability

### Lead contact

Further information and requests for resources and reagents should be directed to and fulfilled by the lead contact, Fabrizio Alberti (f.alberti@warwick.ac.uk).

### Technical contact

Further information on the technical aspects of the protocal should be directed to and fulfilled by the technical contact, Sophie Jin (sophie.jin@warwick.ac.uk).

### Materials availability

All plasmids and fungal strains generated in this study are available from the lead contact without restriction.

### Data and code availability

• The data supporting the current study are available from the corresponding author without restriction.
• This study did not generate code.

## Acknowledgments

This work was supported by a UKRI Future Leaders Fellowship award (MR/V022334/1) to F.A., a 10.13039/501100000268BBSRC Midlands Integrative Biosciences Training Partnership (BB/T00746X/1) scholarship to S.J., and a BBSRC International Partnering Award (BB/X018369/1). The authors thank Noriha Mat Amin and Hamidun Bunawan for providing *Trichoderma koningiopsis* RA6.

## Author contributions

Methodology, investigation, formal analysis, visualization, and writing – original draft, S.J.; writing – review and editing, S.J. and F.A.; funding acquisition, S.J. and F.A.; conceptualization, S.J.; supervision and project administration, F.A.

## Declaration of interests

The authors declare no competing interests.
